# Authors' response (July 16, 2026) to the Letter to the Editor concerning the paper “Psychosocial working conditions and mental well-being of remote and stationary employees: a longitudinal study”

**DOI:** 10.13075/ijomeh.1896.02947

**Published:** 2026

**Authors:** Dorota Żołnierczyk-Zreda, Łukasz Kapica, Andrzej Najmiec, Joanna Kamińska, Joanna Mazur-Różycka, Joanna Bugajska

**Affiliations:** Central Institute for Labour Protection – National Research Institute, Ergonomics Department, Warsaw, Poland

**Keywords:** artificial intelligence, remote work, psychosocial risks, mental well-being, occupational health, work organisation

Dear Editor,

We thank the authors for their thoughtful letter [1] and for drawing attention to the occupational health implications of artificial intelligence (AI) in contemporary work. This is a timely contribution, as AI is becoming an increasingly important element of work organization and may create new psychosocial risks that occupational health research needs to address.

The mechanisms discussed in the letter – task expansion, boundary blurring, rework demands, and continuous low-level engagement with work – help specify how technologies designed to increase efficiency may also change the intensity, timing, and experience of work. They point to a possible gap between task-level efficiency and employees' actual workload or well-being.

The letter shifts attention from remote work itself to a related question: how new technologies may reshape psychosocial working conditions. Emerging evidence suggests that AI-supported work may affect not only task performance, but also interpersonal interactions at work, learning demands, work-life outcomes, and employee well-being. The existing findings, however, are not uniform and may vary depending on the work context and the way AI is introduced and used [2–4].

Our findings [5] suggest that the relationship between work arrangement and mental well-being should be understood in the context of psychosocial working conditions. In our study, some psychosocial aspects of work, including control at work, emotional demands, demands for hiding emotions, role conflict, and trust in co-workers, were assessed more favorably by remote workers than by stationary employees. Previous reviews and meta-analytic evidence also show that the effects of remote work are shaped by autonomy, social support, workload, work-home boundaries, and organizational practices [6–8].

Artificial intelligence should therefore be examined as a factor that may modify psychosocial working conditions. It may support task completion and flexibility, but it may also add new demands, such as work intensification, verification of AI-generated outputs, learning requirements, extended availability, or difficulties in psychological detachment from work. In this sense, the occupational health effects of AI are likely to depend not only on the technology itself, but also on how it changes job demands, job resources, and everyday work practices.

We agree with the authors that the AI efficiency paradox should be treated as a hypothesis requiring further empirical testing. Longitudinal studies are needed to examine how AI use relates to working time, workload, boundary management, recovery, and mental well-being. Framed within the job demands-resources model [9], such studies could help clarify when AI acts as a job resource supporting task performance and flexibility, and when it becomes an additional job demand that contributes to work intensification, impaired recovery, or poorer well-being.
